# Risk Factors for Recurrence of Peptic Ulcer Disease: A Retrospective Study in Tertiary Care Referral Center

**DOI:** 10.7759/cureus.22001

**Published:** 2022-02-07

**Authors:** Yaser M Alsinnari, Mohammed S Alqarni, Meshari Attar, Ziad M Bukhari, Mohammed Almutairi, Faisal M Baabbad, Mohammed Hasosah

**Affiliations:** 1 College of Medicine, King Saud Bin Abdulaziz University for Health Sciences, Jeddah, SAU; 2 Research Office, King Abdullah International Medical Research Center, Jeddah, SAU; 3 Department of Pediatric Gastroenterology, King Abdulaziz Medical City, Ministry of National Guard Health Affairs, Western Region, Jeddah, SAU

**Keywords:** risk factors, h pylori infection, prevalence, recurrence, peptic ulcer disease

## Abstract

Backgrounds

Peptic ulcer disease (PUD) is a common gastrointestinal tract disease characterized by mucosal damage secondary to pepsin and gastric acid secretion. This study evaluated the five-year recurrence rate for patients with PUD and risk factors contributing to PUD relapses.

Methods

From 2016 through 2021, all patients with endoscopy-proved PUD were identified by reviewing medical records (Best-Care system). Possible risk factors including smoking, nonsteroidal anti-inflammatory drugs (NSAIDs), aspirin, alcohol, caffeine, and steroids were analyzed by multivariate analysis. Treatment outcomes, 5-year recurrence rate, and mortality rate were assessed.

Results

Among 223 patients, there were 187 (83.8%) diagnosed with endoscopy-proved PUD and 36 (16.2%) diagnosed with clinical PUD. Among them, 126 (56.5%) patients were males, and the mean age was 62±2 years. The five years recurrence rate of PUD was 30.9%. There was no significant difference in the recurrence rate between the duodenal ulcer (33.3%) and the gastric ulcer (28.8%). By univariate analysis, the use of steroids and NSAID and Helicobacter pylori (H. pylori) infection were potential risk factors for PUD (P < 0.005). The common complication of PUD was gastrointestinal bleeding (34.1%). Patients who had a complicated PUD were associated with a higher rate of recurrence (45.9%) compared to the uncomplicated PUD (19.2%) (P > 0.05).

Conclusion

Our findings demonstrated that the five years recurrence rate of PUD was 30.9%. The use of steroids and NSAID and H. pylori infection were risk factors for recurrence of PUD. PUD places a significant burden on health care systems. Therefore, a multicenter prospective study is needed for effective management to prevent recurrence and complications of PUD.

## Introduction

Peptic ulcers are open sores involving the mucosa through the muscular layer of the stomach or the first portion of the duodenum. Peptic ulcer disease (PUD) is a common pathology in gastroenterology. The incidence rate of PUD ranges from 10% to 19% and impact 4 million people globally each year. One of the hallmarks of peptic ulcer symptoms is abdominal pain, which is usually relieved by food intake or antacids [1]. The occurrence of PUD was recorded at about 1.5 percent to 3 percent [1]. Perforated peptic ulcer (PPU) is a severe complication of PUD, and people with PPU also have severe abdomen with a high risk of long-term illness and fatality [2]. Proton pump inhibitors such as omeprazole, pantoprazole, lansoprazole are widely recognized worldwide as the best pharmacological therapy for both gastric and duodenal ulcers of the stomach [3]

Helicobacter pylori (H. pylori) are the most common cause of peptic ulcers, along with the long-term consumption of nonsteroidal anti-inflammatory drugs (NSAIDs) like sodium naproxen and ibuprofen (Advil, Motrin IB, others). Symptoms of peptic ulcer disease are complex and can include stomach pain, weight loss, nausea, vomiting, and bleeding or perforation of serious diseases [4].

H. pylori eradication helps treat PUD and significantly decreases its recurrence. Effective elimination of H. pylori is by far the most critical factor influencing the relapse of PUD, even though age, drugs, and the prevalence of chronic diseases also impact its recurrence. The risk of relapse of H. pylori infection disease associated with peptic ulcers in adults can be decreased when the bacterium H. Pylori is successfully eradicated. However, the findings differ from study to study. The five-year recurrence rate of PUD is below 5% if there are no potential complications related to NSAIDs after H. pylori eradication [5].

The consumption of Nonsteroidal anti-inflammatory drugs (NSAIDs) and acetylsalicylic acid (ASA), which leads to adverse gastrointestinal events such as peptic ulcers, are becoming more extensive. It is also conceivable that NSAID use is associated with peptic ulcer complications, such as upper gastrointestinal hemorrhage or perforation. Peptic ulcer disease also has a significant burden on health care costs. The overall cost of peptic ulcer disease in the USA, including actual expenses and loss of productivity at work, is estimated to be 5.65 billion USD per year [6]. Consumption therapy (NSAIDs) is well known and is associated with an increased risk of peptic ulcers and non-variceal upper gastrointestinal bleeding. Findings also say that the colon may also be impaired because of excessive use of NSAIDs. Also, the use of these medications is increasing in a growing elderly population. Various studies demonstrated that using proton pump inhibitors (PPIs) reduces upper GI harm and the likelihood of upper GI complications but is not beneficial in avoiding minor intestinal mucosal damage associated with NSAIDs [7]. Chan et al. found that treatment with proton pump inhibitors (PPIs) was more effective in minimizing persistent bleeding over six months than H. pylori elimination alone. In clinical settings, it is essential to recommend concomitant PPIs for all users of NSAIDs who have a PUD medical history of complications [8].

Our study aims to evaluate the five-year recurrence rate for patients with treated peptic ulcer disease and factors contributing to relapse, analyzing PUD complications and their impact along with previous treatment regimen and their effects to determine the efficacy of medications used to prevent a recurrence.

## Materials and methods

This retrospective study included all peptic ulcer patients diagnosed from 2016 to 2021 at King Abdulaziz Medical City in Jeddah, Saudi Arabia. The inclusion criteria were all patients diagnosed with peptic ulcer disease from 2016 to 2021 and patients treated for peptic ulcers. The exclusion criteria were all patients with gastric cancer or comorbidities that affect the gastrointestinal system, such as inflammatory bowel disease. Both inclusion and exclusion criteria were reviewed on the BestCare system, the medical record system at King Abdulaziz Medical City. This research commenced by reviewing the inclusion and exclusion criteria on the BestCare system, and a total of 223 patients were selected for this study.

A datasheet was constructed with demographic variables such as age, gender, comorbidities such as diabetes, hypertension, and psychological histories such as stress, anxiety, and post-traumatic stress disorder (PTSD). Other variables from the treatment regimen were recorded, such as date of diagnosis, type of PUD, proton pump inhibitor (PPI) usage and type of PPI, H2 antagonist and its type, the use of antibiotics and its type, and cytoprotective agents. As well as a diagnostic modality, H. pylori infection, and Zollinger-Ellison syndrome. Risk factors were also recorded in the datasheet, including smoking, NSAID, Aspirin, Alcohol, Caffeine, and steroids. Treatment outcomes were also collected in the datasheet, including side effects, 5-year recurrence rate, date of recurrence, date of first and last visit, complications, and type of complications encountered. Recurrence is noted if PUD reoccurs within five years from previously treated PUD, diagnosed by endoscopy regardless of symptoms. After the diagnosis was made, the patients started the appropriate therapy. Then, the patients were followed for 2 to 4 weeks post-therapy. If the patients developed alarming symptoms (weight loss, upper gastrointestinal bleeding, dysphagia, dysphonia) or patients' symptoms were not improving, they repeated endoscopy. Also, If the patients had any recurrent symptoms, they had a follow-up and repeated endoscopy. Then all data were collected from the medical record software system BestCare.

This article has been posted on a preprint server (https://www.researchsquare.com/article/rs-1184031/v1). The study was in accordance with the Declaration of Helsinki and was ethically approved by the Institutional Review Board (IRB) at King Abdullah International Medical Research Center (KAIMARC), IRB reference number IRBC/1992/21 [9]. All data were then entered and analyzed using Statistical Package for the Social Sciences (SPSS) version 23 (IBM corp., Armonk, NY) [10]. Numerical findings and demographic data collected from the datasheet were computed and presented by frequency and percentage. In addition, a data mean was calculated, and all numerical findings were systematically arranged to provide a quantitative data summary and descriptive data analysis. Categorical variables are presented as frequencies and percentages and continuous variables as standard deviations or medians; interquartile ranges were used when the distributions were skewed. Two-tailed Student's t-test was used to compare means, chi-square test was performed for comparisons of a categorical variable, and multivariate regression was used to analyze continuous variables. Multivariate logistic regression was done with peptic ulcer recurrence as the dependent variable and age, gender, comorbidities, treatment regimen, smoking, NSAID, Aspirin, Alcohol, Caffeine, and steroids. Independent variables. A P-value of <0.05 was considered statistically significant.

## Results

Two hundred twenty-three patients were included in this study. 126 (56.5%) patients were males, and 97 (43.5%) were females. The age of the study population ranged from 5 to 112 years old, with a mean age of 62±21. Of the study population, diabetes was recorded in 115 (51.6%), and hypertension in 136 (61%). PUD was diagnosed using endoscopy and histology in 187 (83.8%) and by clinical diagnosis in 36 (16.2%) patients. The five-year recurrence rate of PUD was 30.9% which was seen in 69 patients. The mean follow-up duration of the study patients was 39±7 months. No significant difference in the recurrence rate between the duodenal (33.3%) and the gastric ulcer (28.8%). Also, there is no significant difference in age and body mass index-site of the ulcer various complications and the mortality rates between the two groups. Multivariate logistic regression was done with peptic ulcer recurrence as the dependent variable and demographic and risk factors as independent variables, which shows no statistical significance. Risk factors were compared against the recurrence rate, as shown in table 1.

**Table 1:** Risk factors associated with 5-years recurrence of Peptic ulcer disease. * other than gastric cancer *chi-square test was used. H. pylori- Helicobacter pylori; NSAID- Nonsteroidal anti-inflammatory drugs

variable	Overall n=223	Recurrence n=69	No recurrence n=154	p value*
Gender				0.5
Male	126	41(59.4%)	85(67.5%)	
Female	97	28(28.9%)	69(71.1%)	
History of depression				0.4
Yes	18	7(38.9%)	11(61.1%)	
No	205	62(30.2%)	143(69.9%)	
H.pylori infection				0.05
Yes	36	16(44.4%)	20(55.6%)	
No	187	53(28.3%)	134(71.7%)	
Cancer*				0.3
Yes	50	13(26%)	37(74%)	
No	173	56(32.4%)	117(67.6%)	
NSAID use				0.01
Yes	49	22(44.9%)	27(55.1%)	
No	174	47(27%)	127(73%)	
Steroid use				0.009
Yes	24	13(54.2%)	11(45.8%)	
No	199	56(28.1%)	143(71.9%)	
Smoking				0.3
Yes	30	7(23.3%)	23(76.7%)	
No	193	62(32.1%)	131(67.9%)	

Various treatment regimens and medication were documented. The association between recurrence and treatment regimen is shown in table 2.

**Table 2:** Treatments associated with 5-years recurrence of peptic ulcer diseases. *chi-square test was used. H. pylori- Helicobacter pylori; PPI- proton pump inhibitors.

variable	Overall n=223	recurrence n=69	No recurrence n=154	P value*
Major Treatment plan				0.02
Avoiding risk factors	15	1(6.7%)	14(93.3%)	
H.pylori eradication	30	15(50%)	15(50%)	
Acid suppression therapy	145	44(30.3%)	101(69.7%)	
Surgical treatment	33	9(27.3%)	24(72.7%)	
PPI use				0.06
Yes	210	68(32.4%)	142(67.6%)	
No	13	1(7.7%)	12(92.3%)	
H2 antagonist use				0.012
Yes	22	12(54.5%)	10(45.5%)	
No	201	57(28.4%)	144(71.6%)	
The use of antibiotics				0.003
Yes	34	18(52.9%)	16(47.1%)	
No	189	51(27%)	138(73%)	
Antacid use (calcium carbonate)				0.2
Yes	34	14(41.2%)	20(58.8%)	
No	189	55(29.1%)	134(70.9%)	

For PPI, the combination of omeprazole and esomeprazole had a rate of recurrence (51.4%) higher than the use of omeprazole (27.1%) or esomeprazole (29.3%) alone (P-value=0.01). Esomeprazole was used when the patients were hospitalized, while omeprazole was used for outpatient treatment The medication used in 22 patients on H2 antagonist therapy was ranitidine. The antibiotic regimen used in the study population was the triple antibiotic H.pylori eradication therapy (with clarithromycin, amoxicillin, and PPI therapy). 98 (43.9%) patients had a complicated PUD. Patients who had a complicated PUD were associated with a higher rate of recurrence (45.9%) compared to the uncomplicated PUD (19.2%) (P-value > 0.05). Different types of complications were seen as shown in table 3. The mortality rate among the study population was 31.4% with no statistically significant association with PUD recurrence. 

**Table 3:** Complication types of Peptic ulcer disease.

Type of complication	n(%)
Gastrointestinal bleeding	76(34.1%)
Perforation	12(5.4%)
Penteration/fistula	3(1.3%)
Gastric outlet obstruction	3(1.3%)
Malignant transformation	5(2.2%)
No complications	124(55.6%)

## Discussion

Peptic ulcer disease does not only cause a large financial burden on the healthcare sector, but its complications, such as perforation, can put patients at increased risk of morbidity and mortality. Our study assessed several risk factors to associate with a 5-year recurrence rate. Three risk factors were identified as statistically significant p-value <0.05, which were H.pylori infection, NSAID, and steroid use. Our study found that H.pylori infection as a risk factor had 0.05 of 36 patients with H.pylori infection 16 (44.4%) had a recurrence, in contrast to patients not having H.pylori infection only 53 our 187 (28.3%) had a recurrence. In accordance with our findings, another study also found a statistically significant difference in H.pylori infected patients vs. non H.pylori infected patients in terms of recurrence where only 1 out of 9 (11.1%) patients without H.pylori had a relapsed gastric ulcer within one year in contrast to 7 out of 18 (38.9%) who had an H.pylori had a relapsed peptic ulcer within one year [11]. They also found that H.pylori infection vs. those not with H.pylori infection had increased the risk of gastric ulcer recurrence and duodenal recurrence (0 vs. 66.7%), respectively.

Another risk factor associated with peptic ulcer recurrence in our study was NSAID use with a p-value of 0.01. 22 out of 49 (44.9%) patients on NSAIDs had a recurrence of peptic ulcers, whereas only 47 out of 174 (27%) patients not on NSAIDs had a recurrence. This result is similar to another study that found that NSAID use had increased PUD recurrence and that increasing doses of NSAIDs had increased the risk of recurrence [12]. In their study, total bare dose (cDDD), the total of allocated defined daily dose (DDD), as compared to those not taking NSAIDs, defined as cDDD of 0. They found that NSAID use vs. nonuse had a matched cohort p-value of 0.004. Nonsteroidal anti-inflammatory drugs (NSAIDs)-induced ulcers are normal in older patients, patients with various medications, patients with chronic conditions, Helicobacter pylori (H. pylori) infection, and background of peptic ulcers. A study recorded that 6.4 percent - 11.8 percent of patients who were administered NSAIDs developed peptic ulcer disease (PUD). Therefore, the recurrence of peptic ulcer disease is strongly associated with NSAIDs. Patients with PUD who have used NSAIDs upon early detection should be screened and treated for H. pylori infection [13].

In our study, steroid use was associated with peptic ulcer recurrence p-value 0.009. Out of 24 patients using steroids, 13 (54.2%) had a recurrence of peptic ulcers. In contrast, only 56 out of 199 (28.1%) had a recurrence for those not on steroids. Similarly, another study found that corticosteroids agents had an increased risk of peptic ulcer bleeding with an odds ratio (OR) of 2.77 with a 95% confidence interval of 0.62-9.62 [14]. They also found that immunosuppressive agents had the highest risk of peptic ulcer bleeding with odds ratios of and 95% confidence interval of 5.83 .88- 22.88. 

The treatment plan and its association to peptic ulcer recurrence within five years was also assessed in our study and found to be statistically significant with a p-value of 0.02. We found that avoiding risk factors had the lowest recurrence rate in contrast to other treatment plans such as eradication of H.pylori, acid suppression therapy, and surgical treatment (6.7%, 50%, 30.3%, 27.3%, respectively). This adds to the emphasis of risk factors identification in patients and addressing them. We also found that H2 antagonist use had a statistically significant impact on peptic ulcer recurrence with a p-value of 0.012. 12 out of 22 (54.5%) patients using H2 antagonists had a recurrence, whereas, in contrast to those who did not receive H2 antagonists, only 57 out of 201 (28.4%) had a recurrence. This suggests a poor outcome in H2 antagonist therapy in the treatment of peptic ulcers. In terms of PPI use and recurrence rate, our study found that it was not statistically significant with a p-value of 0.06. The use of antibiotics was statistically significant, with a p-value of 0.003. Patients on antibiotics had a higher incidence of recurrence vs. those not on antibiotics, 52.9%, and 27%, respectively. This suggests that while antibiotic therapy alone may eradicate H.pylori, it will also put patients at a higher risk of peptic ulcer recurrence in the future.

In terms of complications, our study found that while 55.6% of patients faced no complications, the most common complication faced is gastrointestinal bleeding (GI bleed), 34.1%, which is a life-threatening condition. There is also the issue of Idiopathic peptic ulcer disease (IPUD) which is the occurrence of peptic ulcers without specific causes, such as NSAIDs, H. pylori, and hypergastrinemia. The frequency of IPUD ranges from 1.3 percent to 27 percent due to differences in the rate of H. pylori infection across various locations. The medical effects of IPUD are more critical than PUD due to H. pylori and NSAIDs, and the relapse rate is much higher. In addition, fatalities and risk of repeated bleeding due to idiopathic bleeding ulcers are greater. In a recent study, the 5-year average reinfection rate of IPUD was 24.3 percent which is considerably higher than that for PUD-induced NSAIDs and eliminated H. pylori-positive PUD. The lengthy relapse risk of PUD without H. pylori due to the effects of drugs are believed to be high; furthermore, there is insufficient evidence to support this concern. H. pylori infection can be easily identified in PUD patients using several methods. Therefore, it is critical to analyze the level of H. pylori infection, the most powerful and reliable observable factor, as a significant predictor of PUD relapse. It is well known that infection with H. pylori causes the recurrence of ulcers [4].

Advanced medical therapy and innovative clinical endoscopic procedures have gradually reduced the need for surgical care in patients with PUD over the last 20 years. The most common endoscopic lesions following gastric surgery are marginal ulcers (MUs) occurring in the anastomotic region with a range of frequencies from 6 to 16 percent. The cause of MU is still unknown, although several aspects have been studied, such as the function of H. pylori infection, the significance of surgical anastomosis, and other related risk factors (biliary reflux). Risk factors research that affects the incidence of complications in gastric stumps (cancers, ulcers) are of clinical concern, with a growing number of gastric operations in overweight or early gastric cancer patients [4].

Our study aims to evaluate the recurrence rate for PUD and analyze factors contributing to relapse. Complications of PUD and their impact was analyzed along with previous treatment regimen and their effects to determine the efficacy of medications used in terms of recurrence. Regarding our study's high mortality rate and complications, King Abdelaziz Medical City is a tertiary canter that usually receives patients with multiple co-morbid conditions. Moreover, the medical city also contains an oncology center. These factors make the study population have a high likelihood of developing complications and mortality related to PUD and their co-morbid conditions 

This study has some limitations as it is a retrospective cross-sectional study and is single-center-based, ultimately limiting the results' generalizability. The small sample size of 223 also limits the ability to show a difference between predictive values. Another limitation is the generalization of NSAIDs, as different NSAIDs have various effects on PU, and the same is valid for steroids. One of our significant limitations is that the clinical diagnosis is subjective and passive to clinical judgment error.

## Conclusions

Peptic ulcer disease places a significant burden on health care systems which advocate the need for effective management to prevent recurrence and complications. In this study, three risk factors associated with 5-year peptic ulcer recurrence were found; H. Pylori, NSAID use, and steroid use. NSAIDs and steroids are risk factors commonly prescribed for long-term use. We found that simply avoiding risk factors had the lowest recurrence rate of H. pylori eradication, acid suppression therapy, and surgical treatment. This emphasizes the need to identify risk factors and avoidance for better long-term outcomes. A multicenter prospective study is needed for effective management to prevent recurrence and complications of PUD.
